# MicroRNAs *mir‐184* and *let‐7* alter *Drosophila* metabolism and longevity

**DOI:** 10.1111/acel.12673

**Published:** 2017-09-29

**Authors:** Christi M. Gendron, Scott D. Pletcher

**Affiliations:** ^1^ Department of Molecular and Integrative Physiology and the Geriatrics Center University of Michigan Ann Arbor Michigan 48109 USA

**Keywords:** aging, diet restriction, *let‐7*, *miR‐100*, *miR‐125*, *miR‐184*

## Abstract

MicroRNAs (miRNAs) are small RNA molecules that regulate gene expression associated with many complex biological processes. By comparing miRNA expression between long‐lived cohorts of *Drosophila melanogaster* that were fed a low‐nutrient diet with normal‐lived control animals fed a high‐nutrient diet, we identified *miR‐184*,* let‐7*,* miR‐125*, and *miR‐100* as candidate miRNAs involved in modulating aging. We found that ubiquitous, adult‐specific overexpression of these individual miRNAs led to significant changes in fat metabolism and/or lifespan. Most impressively, adult‐specific overexpression of *let‐7* in female nervous tissue increased median fly lifespan by ~22%. We provide evidence that this lifespan extension is not due to alterations in nutrient intake or to decreased insulin signaling.

## Introduction, results, and discussion

Aging is a complex, dynamic process in which healthy individuals deteriorate. The rate of this decline is influenced by both genetic and environmental factors, many of which are known to slow its progression and increase lifespan. These effects often manifest quite rapidly. Changes in diet or social conditions, for example, altered mortality rates in fruit flies (*Drosophila melanogaster*) and nematode worms (*Caenorhabditis elegans*) in as little as 12 h and were accompanied by concurrent alterations in physiology and behavior (Mair *et al*., 2003; Smith *et al*., 2008; Gendron *et al*., 2014).

MicroRNA (miRNA) molecules play an important role in the dynamic regulation of a wide variety of complex physiological and pathophysiological processes, and there is growing evidence of their importance in metabolism and aging (Boehm & Slack, 2006; Inukai & Slack, 2013). Overexpression of miRNA *lin‐4* in *C. elegans* significantly increased worm lifespan in a manner that was dependent on both the insulin signaling transcription factor *daf‐16/FOXO* (a known metabolic regulator) and on the heat shock transcription factor *hsf‐1* (Boehm & Slack, 2005). *miR‐34* (Yang *et al*., 2013), *miR‐71* (de Lencastre *et al*., 2010; Boulias & Horvitz, 2012), *miR‐80* (Vora *et al*., 2013), *miR‐238*,* miR‐239*, and *miR‐246* (de Lencastre *et al*., 2010) have also been shown to influence nematode lifespan, with *miR‐71*,* miR‐80*, and *miR‐239* showing dependence on insulin signaling to modulate lifespan. In flies, *miR‐34* (Liu *et al*., 2012) and *miR‐277* (Esslinger *et al*., 2013) have been shown to affect lifespan; *miR‐34* overexpression increased lifespan whereas *miR‐277* overexpression shortened it. While the ability of *miR‐34* to influence metabolism is yet to be investigated, *miR‐277* has been shown to modulate target of rapamycin (TOR) signaling.

To identify new miRNAs that mediate aging in *Drosophila,* we used dietary ‘switch’ experiments. In these experiments, animals switched from a standard to a low‐nutrient environment experience a rapid drop in their age‐specific mortality rate, whereas those moved to a high‐nutrient diet experience mortality increases (Mair *et al*., 2003). We isolated small RNAs from female *Drosophila melanogaster* 3 days after the diet switch. Deep sequencing identified *let‐7*,* miR‐184*,* miR‐34*, and *miR‐8* as differentially expressed between the two diets (Fig. S1A). *miR‐8* abundance appeared to be increased in fully fed conditions while the other three were more abundant in conditions of diet restriction, suggesting that their expression may suppress aging.

**Figure 1:**
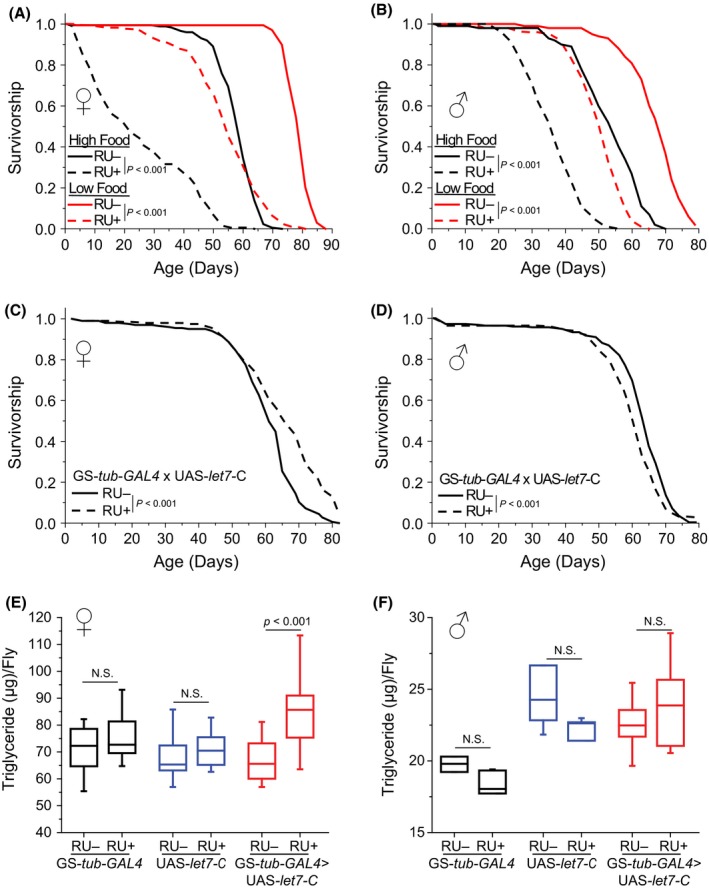
Adult‐specific, ubiquitous overexpression of *miR‐184* and the *let‐7‐C* alters fly lifespan and metabolism. (A) Ubiquitous *miR‐184* overexpression in females drastically shortened lifespan, regardless of food type (*N* = 174 flies for high food RU−, 171 flies for high food RU+, 167 flies for low food RU−, and 170 flies for low food RU+). (B) Ubiquitous *miR‐184* overexpression in males drastically shortened lifespan, regardless of food type (*N* = 192 flies for both high food RU− and RU+, 191 flies for low food RU−, and 194 flies for low food RU+). (C) Ubiquitous overexpression of *let‐7‐C* significantly increased both the median (from 61 to 66 days) and maximal (from 75 to 83 days in the 10% longest lived) lifespan of female flies kept on a high‐nutrient diet (*N* = 201 flies for RU− food and 197 flies for RU+ food). (D) Ubiquitous overexpression of *let‐7‐C* significantly decreased male lifespan in flies kept on a high‐nutrient diet (*N* = 249 flies for RU− food and 244 flies for RU+ food). (E) Ubiquitous overexpression of *let‐7‐C* significantly increased triglycerides in female flies (*N* = 50 flies per genotype/food treatment). (F) Ubiquitous overexpression of *let‐7‐C* had no effect on male triglyceride amounts (*N* = 25 flies for each GS‐*tub‐GAL4* and UAS‐*let7‐C* food treatment, *N* = 50 flies for each GS‐*tub‐GAL4 *>** UAS‐*let7‐C* food treatment).

Given that *miR‐34* is known to influence lifespan and neurodegeneration (Liu *et al*., 2012), we focused our investigation on *miR‐184* and *let‐7*. To determine whether increased *miR‐184* expression promotes lifespan, we used the Gene Switch (GS) system to induce *miR‐184* expression broadly in the adult animal (GS‐*tubulin‐GAL4 *>* *UAS‐*miR‐184*). Flies fed the transcriptional activator RU‐486 for 3 days showed a fourfold increase in *miR‐184* expression compared to isogenic flies fed vehicle (Fig. S1B). In male and female flies, ubiquitous *miR‐184* overexpression severely reduced lifespan, independent of diet, and failed to inhibit lifespan extension through dietary restriction (Fig. 1A and B). These data suggest that adult‐specific *miR‐184* overexpression is deleterious regardless of diet.

Aging and metabolic homeostasis are often linked (Finkel, 2015). To exemplify, dietary restriction not only increases lifespan, but also increases fat (Kapahi *et al*., 2016). Metabolic state is often indicated by triglyceride (TAG) abundance, the primary storage lipid in the fly. We therefore examined the effect of *miR‐184* overexpression on the abundance of triglyceride (TAG). We observed no effect of *miR‐184* overexpression on TAG abundances after 3 days of RU‐486 feeding, when >92% of the flies are still alive (Fig. S1C and D).

We next asked whether *let‐7* influences aging. In flies, *let‐7* is co‐transcribed as part of the *let‐7‐complex* (*let‐7‐C*), which is a single RNA transcript comprised of *miR‐100, miR‐125*, and *let‐7* (Fig. S2A) (Pasquinelli *et al*., 2000). We found that broad *let‐7‐C* overexpression significantly increased both the median and maximal lifespan of females kept on a high‐nutrient diet (Fig. 1C), as well as the median lifespan of female flies kept in low‐nutrient conditions (Fig. S2B). Female lifespan was also extended using a second putatively ubiquitous GS driver (Fig. S2C) but not in control crosses (Fig. S2D,E, and F). Male flies overexpressing *let‐7‐C* were modestly, but significantly, shorter‐lived (Fig. 1D), revealing a sexually dimorphic effect. *Let‐7‐C* overexpression also led to increased TAG stores in female flies but not males (Fig. 1E and F). Flies lacking *let‐7‐C* are known to experience severe developmental lethality with a small percentage of escapers that exhibit a shortened lifespan (Caygill & Johnston, 2008). We found that surviving female adults had less TAG abundance, which is consistent with the notion that *let‐7‐C* expression promotes TAG storage (Fig. S3A). Of note, the fecundity of *let‐7‐C* overexpression flies was similar to control flies, establishing that the increased lifespan seen in females does not require changes in reproduction (Fig. S3B).

Attempts to identify a single tissue in which adult‐specific overexpression of the *let‐7‐C* is sufficient to extend lifespan were not successful. Overexpression in the fat body (using the GS‐S_1_106‐*GAL4* driver line), nervous system (using the GS‐*elav‐GAL4* driver line), or gut (using the GS‐TIGS2‐*GAL4* driver line) had no effect on female lifespan (Fig. S4A,B, and C). These data suggest that *let‐7‐C* overexpression is required in a currently untested tissue type and/or a combination of tissues to promote lifespan extension.

**Figure 2:**
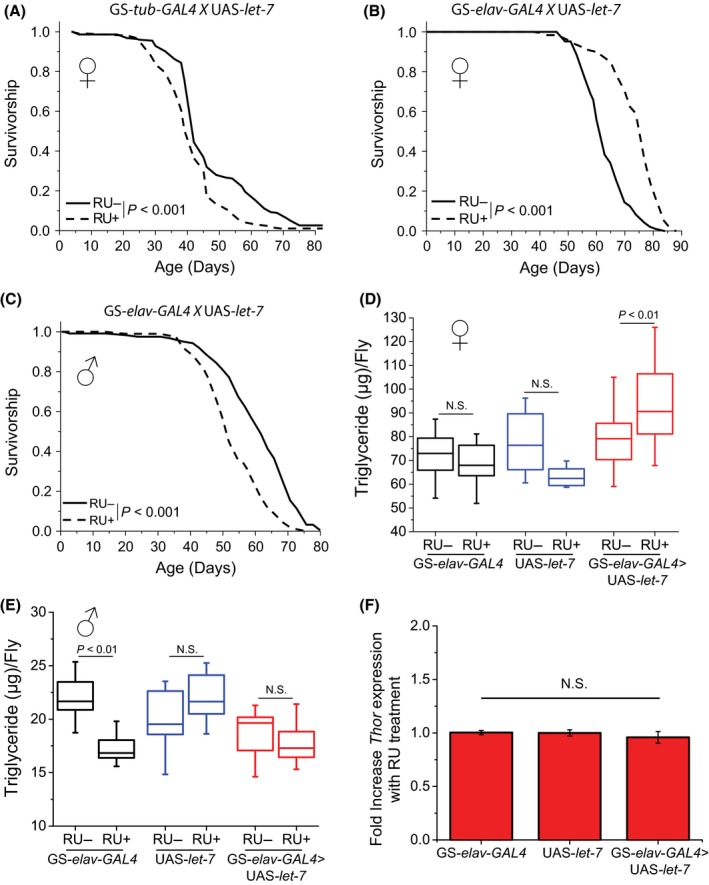
Adult‐specific, neuronal *let‐7* overexpression increases female lifespan and TAG. (A) Ubiquitous *let‐7* overexpression in females significantly decreased female lifespan (*N* = 225 flies for both food treatments). (B) Neuronal *let‐7* overexpression significantly increased female lifespan (*N* = 167 flies for RU− food and 61 for RU+ food), while having the opposite effect in males (C; *N* = 101 flies for RU− food and 123 flies for RU+ food). (D) Neuronal *let‐7* overexpression significantly increased female TAG levels (*N* = 50 flies per genotype/food treatment). (E) Neuronal *let‐7* overexpression had no significant effect on male TAG levels (*N* = 50 flies per genotype/food treatment). (F) *4E‐BP* mRNA levels are unaltered in females with neuronal *let‐7* overexpression (*N* = 50 flies per genotype/food treatment).

To identify which components of the *let‐7‐C* are responsible for extended female lifespan and increased TAG, we ubiquitously overexpressed individual miRNAs. The specificity of our transgenic constructs was confirmed by qPCR (Fig. S5A). Surprisingly, broad overexpression of individual *let‐7‐C* members either significantly decreased lifespan (*let‐7* and *miR‐125;* Fig. 2A and Fig. S5B) or had no effect (*miR‐100*; Fig. S5C). The negative effect of *miR‐125* overexpression on lifespan was unexpected given that *Drosophila miR‐125* is the homologue of *C. elegans lin‐4,* a miRNA that was previously demonstrated to increase worm lifespan when overexpressed (Boehm & Slack, 2005). These data indicate that *miR‐125* and *lin‐4* have different functions between species. Ubiquitous overexpression of *let‐7* significantly increased TAG levels (Fig. S5D), whereas ubiquitous overexpression of *miR‐125* or *miR‐100* did not (Fig. S5E and F), suggesting that the increased TAG seen with ubiquitous *let‐7‐C* overexpression is due to *let‐7* itself.

Although overexpression of *let‐7‐C* in specific tissues was unable to increase lifespan, it is possible that the tissue‐specific effects of miRNAs of opposite valence combined to zero effect. *let‐7* itself has been implicated in both neuronal proliferation and differentiation (Meza‐Sosa *et al*., 2012). We therefore asked whether neuronal overexpression of *let‐7,* or other members of the *let‐7‐C,* would affect longevity and/or metabolism. We found that *let‐7* overexpression in neurons caused a significant increase in female median (22%) and maximum (14%) lifespan (Fig. 2B). In contrast, overexpression of *miR‐125* in neurons reduced lifespan, while *miR‐100* overexpression had no effect (Fig. S6A and B). Mirroring the sex‐specific effects of *let‐7‐C* overexpression, we found that *let‐7* overexpression in male neurons significantly decreased lifespan (Fig. 2C). Female TAG levels were significantly elevated when *let‐7* alone was overexpressed in neurons only (Fig. 2D). Male TAG levels may also be increased given that the significant TAG decrease seen in the driver‐only control disappears with *let‐7* overexpression (Fig. 2E).

Next, we investigated whether lifespan extension from neuronal *let‐7* overexpression was caused by either self‐imposed diet restriction or decreased insulin signaling. We used the FLIC (*F*ly *L*iquid *I*nteraction *C*ounter; Ro *et al*., 2014) to measure feeding behavior and found that neuronal *let‐7* overexpression did not alter total feeding interactions, implying that both transgenic and control flies taste and eat food with similar duration and frequency (Fig. S7). QPCR data examining the effect of neuronal *let‐7* overexpression on systemic *Thor (4E‐BP)* mRNA levels revealed no significant changes (Fig. 2F), suggesting that neuronal *let‐7* overexpression increases lifespan in a manner that is independent of systemic insulin signaling.

The mechanism through which neuronal *let‐7* expression influences metabolism and lifespan remains to be determined. *let‐7* is expressed in different brain neuropil regions, including the optic lobes, the antennal lobes (homologous to the mammalian olfactory bulb), the central complex (involved in locomotor and visual behavior), and the mushroom body (homologous to the mammalian hypothalamus) (Kucherenko *et al*., 2012). Furthermore, *let‐7* is predicted to regulate ~48 different mRNA molecules [PICTAR‐FLY; (Grun *et al*., 2005)], many of which are neuronally expressed (76% of profiled mRNA targets). None of these targets are currently known to be involved in fat accumulation and/or turnover. A candidate lifespan screen overexpressing *let‐7* in specific sets of neurons that express predicted *let‐7* targets (such as *Dh44*‐ and *ETHR*‐expressing neurons) failed to implicate specific targets (Fig. S8).

Herein, we have uncovered a new role for the conserved miRNA *let‐7* in aging. While previous work has suggested that adult maintenance of *let‐7‐C* expression is required for healthy male lifespan (Chawla *et al*., 2016), this is the first work demonstrating that *let‐7‐C* overexpression is sufficient to increase normal lifespan. Furthermore, the ability of *let‐7‐C* and neuronal *let‐7* to increase lifespan and alter metabolism is sexually dimorphic, showing significant increases in female lifespan and TAG while having little or even the opposite effect in males. This result may not be surprising given the role of *let‐7* in male germline stem cell behavior (Toledano *et al*., 2012) and in cell‐specific sexual identity (Fagegaltier *et al*., 2014).

## Funding

This work was supported by the following sources: the National Institute of Health grants R01AG030593, R01GM102279, and RO1AG023166 from the National Institute on Aging (NIA).

## Conflict of interest

The authors declare that they do not have any conflict of interests.

## Author contributions

C. Gendron designed the experiments, performed the experiments, and wrote the article. S. Pletcher designed the experiments and wrote the article.

## Supporting information


**Appendix S1**. Methods.

**Fig. S1** Identification of miRNA that are altered through diet, and analysis of *miR‐184* overexpression flies. (A) Several miRNA are altered in flies when given either a high‐nutrient diet compared to those a low‐nutrient diet. Here, we highlight 4 miRNA that appeared to show some diet dependency: *let‐7*,* miR‐8*,* miR‐34*, and *miR‐184*. (B) qPCR of GS‐*tubulin‐GAL4 *>* * UAS‐*miR‐184* flies show that feeding RU‐486 induces a 4‐fold increase in *miR‐184* levels (N = 10 flies per food type). Ubiquitous overexpression of *miR‐184* has no effect on TAG levels in females (C) or in males (D). In panel (C), N = 50 female flies for genotype/food treatment; in panel (D) N = 30 male flies for genotype/food treatment.
**Fig. S2** Adult‐specific *let‐7‐C* overexpression increases female lifespan, regardless of diet or type of ubiquitous driver. (A) Cartoon of the *let‐7‐complex*. All 3 miRNA of the *let‐7‐C* (*miR‐100*,* let‐7*, and *miR‐125*) are transcribed as a polycistronic mRNA molecule. (B) Adult‐specific *let‐7‐C* overexpression using the GS‐*tubulin‐GAL4* driver significantly increases female fly lifespan, regardless of diet (N = 201 flies for high food RU‐, 197 flies for high food RU+, 198 flies for low food RU‐, and 199 flies for low food RU+). (C) Adult‐specific *let‐7‐C* overexpression using the GS‐*daughterless‐GAL4* driver also significantly increases female fly lifespan (N = 170 flies for RU‐ food and 173 flies for RU+ food). Control crosses consisting of the GS‐*tubulin‐GAL4* driver (D; N = 200 flies for RU‐ food and 196 flies for RU+ food), the GS‐*daughterless‐GAL4* driver (E; N = 169 flies for both food types), or UAS‐*let7‐C* (F; N = 195 flies for RU‐ food and 185 flies for RU+ food), with or without RU‐486 feeding, has no significant effect on lifespan.
**Fig. S3** TAG is significantly decreased in *let‐7‐C* mutant female flies (A) and *let‐7‐C* overexpression has no effect on fecundity (B). In (A), N = 50 flies for both genotypes. (B) The number of eggs laid from female GS‐*tubulin‐GAL4 *>* * UAS‐*let7‐C* were counted every day for 7 days (N = 15 flies per food treatment).
**Fig. S4** Adult‐specific overexpression of the *let‐7‐C* in the fat body (A), the nervous system (B), or the gut (C) has no significant effect on female lifespan. For GS‐*S*_*1*_ *106‐GAL4* X UAS‐*let7‐C*, N = 173 flies for RU‐ food and 174 flies for RU+ food. For GS‐*elav‐GAL4* x UAS‐*let7‐C*, N = 171 flies for RU‐ food and 174 flies for RU+ food. For GS‐TIGS2‐*GAL4* x UAS‐*let7‐C*, N = 175 flies for RU‐ food and 173 flies for RU+ food.
**Fig. S5** Characterizing the overexpression of individual *let‐7‐C* members on lifespan and TAG. (A) QPCR of each fly genotype used to overexpress *miR‐100*,* let‐7*, or *miR‐125* (N = 20 flies each per genotype/food treatment). (B) Ubiquitous, adult‐specific overexpression of *miR‐125* significantly decreases female lifespan (N = 251 flies for RU‐ food and 250 flies for RU+ food). (C) Ubiquitous, adult‐specific overexpression of *miR‐100* has no effect on female lifespan (N = 222 flies for RU‐ food and 218 for RU+ food). (D) Ubiquitous, adult‐specific overexpression of *let‐7* significantly increases TAG levels (N = 50 flies per genotype/treatment with the exception of GS*‐tub‐GAL4 *>* * UAS‐*let‐7* on RU‐ food, where N = 40 flies). There is no significant effect of *miR‐125* (E; N = 150 flies per genotype/treatment with the exception of UAS‐*miR‐125* where N = 50 flies/treatment) or *miR‐100* (F; N = 50 flies per genotype/treatment) overexpression on TAG.
**Fig. S6** Neuronal overexpression of *miR‐125* or *miR‐100* does not increase fly lifespan. (A) Neuronal overexpression of *miR‐125* significantly decreases female lifespan (N = 172 flies for RU‐ food and 171 flies for RU+ food). (B) Neuronal overexpression of *miR‐100* has no effect on female lifespan (N = 173 flies for RU‐ food and 172 flies for RU+ food).
**Fig.S7** Overexpressing *let‐7* in neurons does not affect female feeding. N = 12 female flies per genotype/treatment.
**Fig. S8** *Let‐7* overexpression in specific neuronal subpopulations, *Dh44*‐expressing (A) or *ETHR*‐expressing (B) neurons, does not significantly increase female lifespan. In panel (A), N = 197 flies for *Dh44‐GAL4 *>* * UAS‐*let‐7* and *yw* x UAS‐*let‐7*; N = 198 flies for *Dh44‐GAL4* x *yw*. In panel (B), N = 199 flies for *ETHR‐GAL4 *>* * UAS‐*let‐7* and *w* ^*1118*^ x UAS‐*let‐7*; N = 200 flies for *ETHR‐GAL4* x *yw*.
