# Supplementary material for: MicroRNAs mir‐184 and let‐7 alter Drosophila metabolism and longevity
Source: Aging Cell. 2017 Sep 29;16(6):1434–8. doi: 10.1111/acel.12673 (PMC5676060; doi:10.1111/acel.12673)
Supplement: Supplementary file 1 — Appendix S1. Methods. [file ACEL-16-1434-s001.docx]

**Supplementary Methods and Figure Legends**

**Gendron and Pletcher**

**MicroRNAs *miR-184* and *let-7* Alter *Drosophila* Metabolism and Longevity**

**Appendix S1. Methods**

***Experimental Procedures***

**Fly Stocks**

The *yw*, Canton S, *Dh44-GAL4*, and *ETHR-GAL4* lines were obtained from the Bloomington Drosophila Stock Center (Bloomington, Indiana). The *w^1118^* line was obtained from the VDRC (Vienna, Austria). The GS-*tubulin-GAL4*, GS-*TIGS-GAL4*, and GS-*S_1_106-GAL4* lines were graciously provided by Prof. R. Davis (The Scripps Research Institute, Jupiter, FL). The GS-*Daugherless-GAL4* line was kindly provided by Prof. Linda Partridge (UCL, London, UK). The GS-*elav-GAL4* line used herein contains 3 copies of the *elav-GAL4* insertion (one on the second and two on the third chromosome) and was created using standard genetic *Drosophila* techniques in our laboratory by Dr. Brian Chung. The UAS-*miR-184* line was kindly provided by Prof. Ulrike Gaul (University of Munich, Munich, Germany). The UAS-*let-7-complex*, *let-7*^GK1^, and *let-7-C^K01/GK1^*; P{W8, *let7C^Δlet-7^*} lines were graciously provided by Prof. Victor Ambros (University of Massachusetts, Worcester, Massachusetts). The UAS-*let-7* and UAS-*miR-125* lines were generously provided by Prof. Laura Johnston (Columbia University, New York, NY). The UAS-*miR-100* line was created by the authors using standard genetic *Drosophila* techniques as detailed below.

**Deep Sequencing**

Age-matched embryos from a wild type lab stock, Canton S, were aliquoted in a density-controlled manner onto standard fly media. After eclosion, the flies were allowed to mate for two days on 10% sugar/yeast (SY) food. Female flies were then collected and placed onto either SY5% or SY15% food for 72 hours, upon which time the flies were harvested and frozen. Total RNA was extracted using TRIzol (ThermoFisher Scienctific, Inc.) according to the manufacturer’s directions. Deep sequencing analysis was performed at the HGSC, Baylor College of Medicine (Houston, TX). In the plot shown, each dot represents one miRNA; the X- and Y- axis represents the fraction of one miRNA/total miRNA that was sequence for that food type. *miR-1* levels were subtracted from the total miRNA reads in both food types since this miRNA accounted for over ≥68% of the total reads.

**Creation of the *UAS-miR*-100 Fly**

The following primers were used to amplify the *miR-100* gene (italic letters denotes the restriction sites added to each primer; capital letters denotes the *mir-100* sequence):
Forward primer: 5’ cttagc*agatct*CCGCCCTAATGATTTCTTAT 3’
Reverse primer: 5’ gt*gggtacc*TTATCCTTACTCCGCCATTT 3’

After PCR amplification, the product was cloned into a pUAST-attb vector and submitted for sequencing analysis. The *miR-100-*containing vector was then sent to Best Gene, Inc. (Chino Hills, CA) for injection into attp-containing *Drosophila* embryos. Males with the UAS-*miR-10*0 construct insertion were then backcrossed into the *w^1118^* genetic background for a minimum of 8 generations.

**Survivorship assays**

All of the flies used were given a similar larval environment and were aged matched. To accomplish this, embryos laid from each cross within a 16-24 hour period were collected from grape agar plates using PBS. The same number of embryos were then added to bottles containing standard fly media and raised at 25°C in a humidified incubator with 12:12 light:dark cycles. Emerged flies were collected within a 24 hour period and allowed to mate for two days on standard media. After 48 hours, the flies were lightly gassed using CO_2_ and sorted by gender (20-25 per vial) onto the experimental food. The experimental food consisted of either 5% sugar/yeast (low-nutrient food) or 15% sugar/yeast (high-nutrient food) containing 200 μM RU-486 (RU+) or ethanol vehicle (RU-). A minimum of 8 independent vials were set up per gender/experimental treatment. The flies were housed in the 25°C humidified incubator with 12:12 light:dark cycles for their entire lifespan. Flies were given fresh food every 2-3 days and the number of dead recorded using DLife software (Linford et. al. 2013). All lifespans were analyzed using a Log-rank test.

**Triglyceride assays**

After the aged-matched, mated flies had been on the food for 3 days (for UAS-*miR-184* crosses) or 10 days (all other genotypes), the flies were collected (five flies per sample) and homogenized in 150 μl PBS/0.05% Triton-X. Ten μl of the resulting homogenate (diluted 1:20 in PBS/0.05% Triton-X for females or left undiluted for males) was added to 150 μl of Infinity Triglyceride Reagent (Thermo Electron Corp.) for a 10 minute incubation at 37°C. TAG concentrations were determined by the absorbance at 520nm and estimated using a known TAG standard. Average TAG values were based on 6-10 independent biological replicates from multiple vials. All TAG data are plotted as box plots with the middle line representing the median, the box representing the 25^th^ and 75^th^ percentiles, and the whiskers representing the spread of the data. For statistical analysis, we used a 2-way ANOVA with a Sidak’s multiple comparisons post-test.

**Fecundity assay**

All flies were given a similar larval environment as described above and virgin female GS-*tub-GAL4* > UAS-*let-7-C* flies were collected upon eclosion. After 2 days (to confirm that the flies were virgin), 3 virgin females were placed with 9 male flies in a vial (5 vials total for each geno-type) containing 15% sugar/yeast food with 200 μM RU-486 (RU+) or ethanol vehicle (RU-). The number of eggs laid were counted every day over a period of 7 days. The data were analyzed using a 2-way ANOVA with a Sidak’s multiple comparisons post-test.

**QPCR**

All flies for qPCR analysis were flash frozen in liquid nitrogen for storage at -80°C. Total mRNA was extracted for each genotype in FastPrep Lysing Matrix D tubes (MP Biomedicals) using 1 ml cold TRIzol (ThermoFisher Scienctific, Inc.) according to the manufacturer’s directions. The RNA-containing fraction (the top 450μl) was then removed by pipet to a fresh, autoclaved microcentrifuge tube for RNA precipitation using 50μl 3M sodium acetate in DEPC water and 500μl 100% isopropanol. After 30 minutes at -80°C, the RNA pellet was collected by spinning at 13,000rpm in a benchtop centrifuge. The pellet was washed 2X using 70% ethanol in DEPC water and then dissolved in 20μl DEPC water for spectrophotometer analysis at 260nm. Equal amounts of RNA from each sample were then subjected to reverse transcription using either the TaqMan MicroRNA Reverse Transcription Kit for miRNA amplification (Applied Biosystems) or the SuperScript III RT enzyme and protocol (Invitrogen) according to the manufacturer’s directions. The subsequent cDNA was then used for qPCR analysis in a StepOne Plus 96-well Thermocycler (Applied Biosystems) using either the TaqMan MicroRNA Assay System specified for *miR-184, let-7, miR-125, or miR-100* miRNA amplification (Applied Biosystems) or SYBR Green PCR Master Mix (Applied Biosystems) according to the manufacturer’s instructions. The primer sequences used to amplify *Thor* (*4E-BP*) are as follows: Forward primer: 5’ CGAACAGCCAACGGTGAACA 3’; Reverse primer: 5’ TTCCGCTGGACGTGTAAGCA 3’. The data were analyzed using a Student’s t-test.

**Feeding (FLIC) assay**

The details for this assay are described in Ro, *et al*. (PLOS One 2015).

***Supplemental Figure Legends***

**Supplemental Figure 1: Identification of miRNA that are altered through diet, and analysis of *miR-184* overexpression flies.** (A) Several miRNA are altered in flies when given either a high-nutrient diet compared to those a low-nutrient diet. Here, we highlight 4 miRNA that appeared to show some diet dependency: *let-7*, *miR-8*, *miR-34*, and *miR-184*. (B) qPCR of GS-*tubulin-GAL4* > UAS-*miR-184* flies show that feeding RU-486 induces a 4-fold increase in *miR-184* levels (N = 10 flies per food type). Ubiquitous overexpression of *miR-184* has no effect on TAG levels in females (C) or in males (D). In panel (C), N = 50 female flies for genotype/food treatment; in panel (D) N = 30 male flies for genotype/food treatment.

**Supplemental Figure 2: Adult-specific *let-7-C* overexpression increases female lifespan, regardless of diet or type of ubiquitous driver.** (A) Cartoon of the *let-7-complex*. All 3 miRNA of the *let-7-C* (*miR-100*, *let-7*, and *miR-125*) are transcribed as a polycistronic mRNA molecule. (B) Adult-specific *let-7-C* overexpression using the GS-*tubulin-GAL4* driver significantly increases female fly lifespan, regardless of diet (N = 201 flies for high food RU-, 197 flies for high food RU+, 198 flies for low food RU-, and 199 flies for low food RU+). (C) Adult-specific *let-7-C* overexpression using the GS-*daughterless-GAL4* driver also significantly increases female fly lifespan (N = 170 flies for RU- food and 173 flies for RU+ food). Control crosses consisting of the GS-*tubulin-GAL4* driver (D; N = 200 flies for RU- food and 196 flies for RU+ food), the GS-*daughterless-GAL4* driver (E; N = 169 flies for both food types), or UAS-*let7-C* (F; N = 195 flies for RU- food and 185 flies for RU+ food), with or without RU-486 feeding, has no significant effect on lifespan.

**Supplemental Figure 3: TAG is significantly decreased in *let-7-C* mutant female flies (A) and *let-7-C* overexpression has no effect on fecundity (B).** In (A), N = 50 flies for both genotypes. (B) The number of eggs laid from female GS-*tubulin-GAL4* > UAS-*let7-C* were counted every day for 7 days (N = 15 flies per food treatment).

**Supplemental Figure 4: Adult-specific overexpression of the *let-7-C* in the fat body (A), the nervous system (B), or the gut (C) has no significant effect on female lifespan.** For GS-*S_1_106-GAL4* X UAS-*let7-C*, N = 173 flies for RU- food and 174 flies for RU+ food. For GS-*elav-GAL4* x UAS-*let7-C*, N = 171 flies for RU- food and 174 flies for RU+ food. For GS-TIGS2-*GAL4* x UAS-*let7-C*, N = 175 flies for RU- food and 173 flies for RU+ food.

**Supplemental Figure 5: Characterizing the overexpression of individual *let-7-C* members on lifespan and TAG.** (A) QPCR of each fly genotype used to overexpress *miR-100*, *let-7*, or *miR-125* (N = 20 flies each per genotype/food treatment). (B) Ubiquitous, adult-specific overexpression of *miR-125* significantly decreases female lifespan (N = 251 flies for RU- food and 250 flies for RU+ food). (C) Ubiquitous, adult-specific overexpression of *miR-100* has no effect on female lifespan (N = 222 flies for RU- food and 218 for RU+ food). (D) Ubiquitous, adult-specific overexpression of *let-7* significantly increases TAG levels (N = 50 flies per genotype/treatment with the exception of GS*-tub-GAL4* > UAS-*let-7* on RU- food, where N = 40 flies). There is no significant effect of *miR-125* (E; N = 150 flies per genotype/treatment with the exception of UAS-*miR-125* where N = 50 flies/treatment) or *miR-100* (F; N = 50 flies per genotype/treatment) overexpression on TAG.

**Supplemental Figure 6: Neuronal overexpression of *miR-125* or *miR-100* does not increase fly lifespan.** (A) Neuronal overexpression of *miR-125* significantly decreases female lifespan (N = 172 flies for RU- food and 171 flies for RU+ food). (B) Neuronal overexpression of *miR-100* has no effect on female lifespan (N = 173 flies for RU- food and 172 flies for RU+ food).

**Supplemental Figure 7: Overexpressing *let-7* in neurons does not affect female feeding.** N = 12 female flies per genotype/treatment**.**

**Supplemental Figure 8: *Let-7* overexpression in specific neuronal subpopulations, *Dh44*-expressing (A) or *ETHR*-expressing (B) neurons, does not significantly increase female lifespan.** In panel (A), N = 197 flies for *Dh44-GAL4* > UAS-*let-7* and *yw* x UAS-*let-7*; N = 198 flies for *Dh44-GAL4* x *yw*. In panel (B), N = 199 flies for *ETHR-GAL4* > UAS-*let-7* and *w^1118^* x UAS-*let-7*; N = 200 flies for *ETHR-GAL4* x *yw*.
