# Supplementary figures and images for: MicroRNAs mir‐184 and let‐7 alter Drosophila metabolism and longevity
Source: Aging Cell. 2017 Sep 29;16(6):1434–8. doi: 10.1111/acel.12673 (PMC5676060; doi:10.1111/acel.12673)

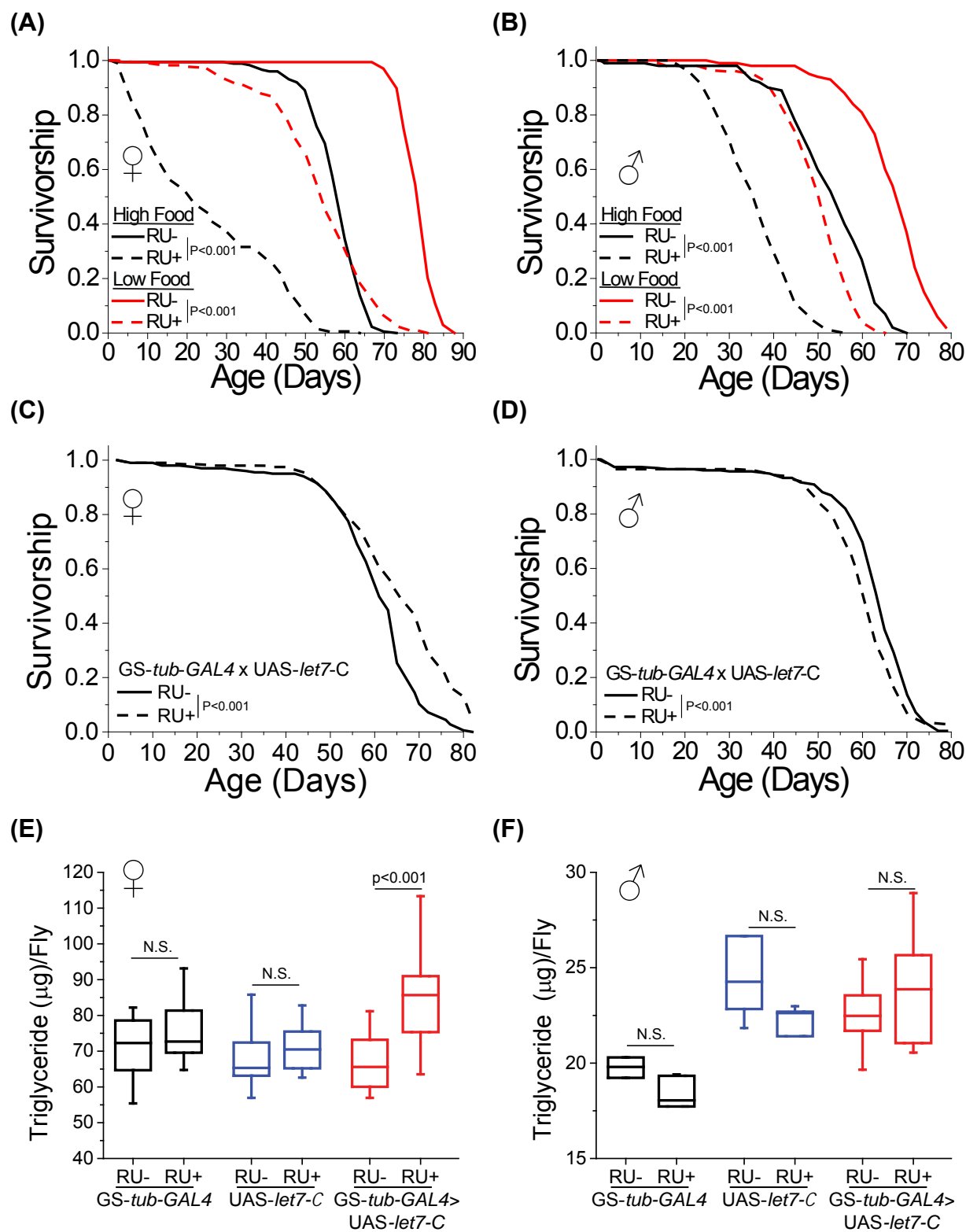

**Figure 1**

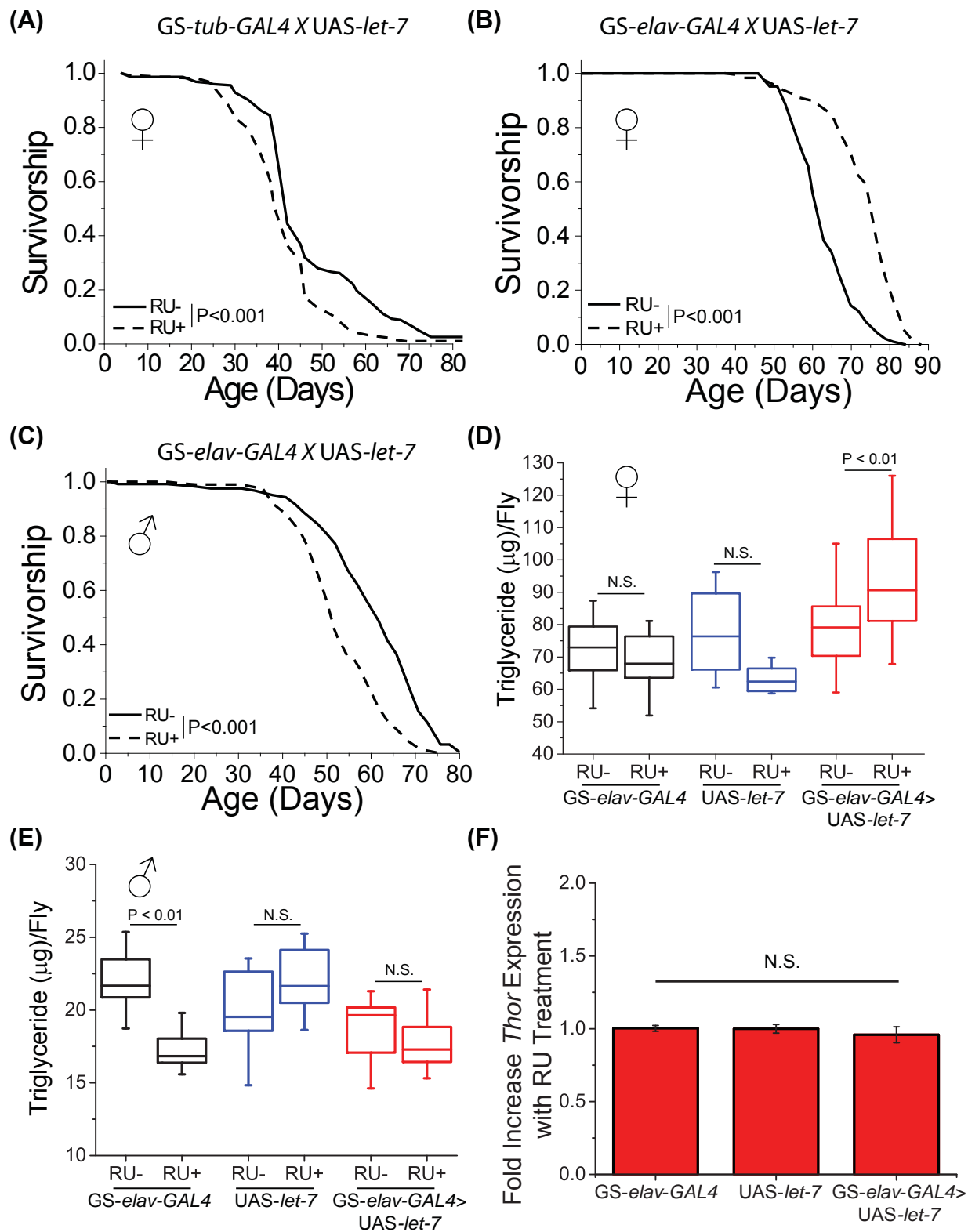

**Figure 2**

(A)

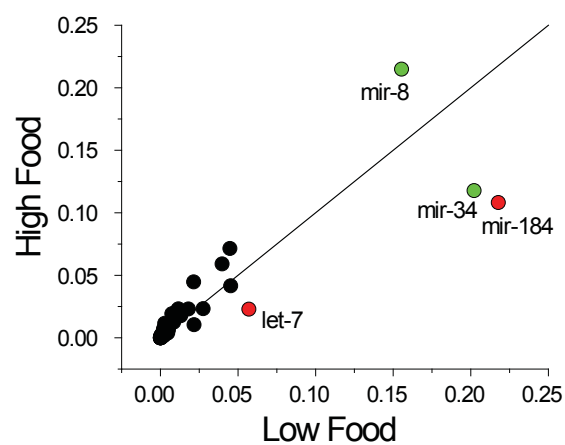

(B)

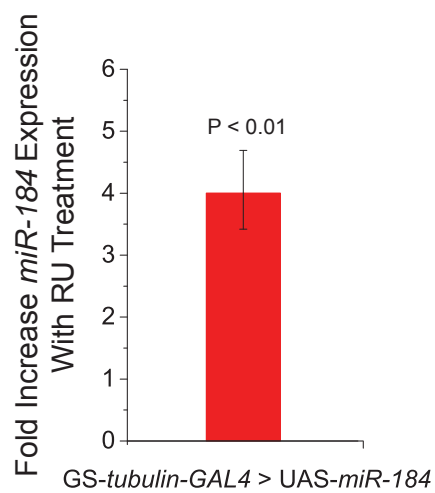

(C)

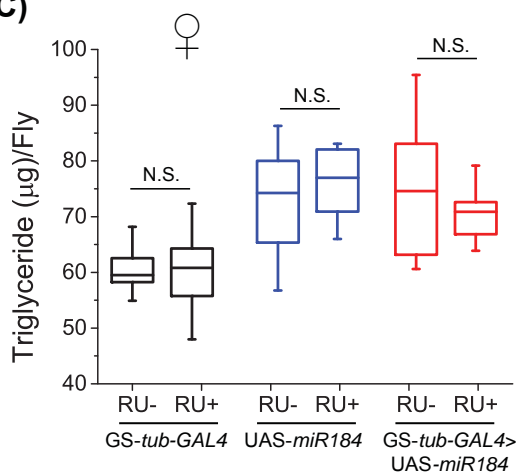

(D)

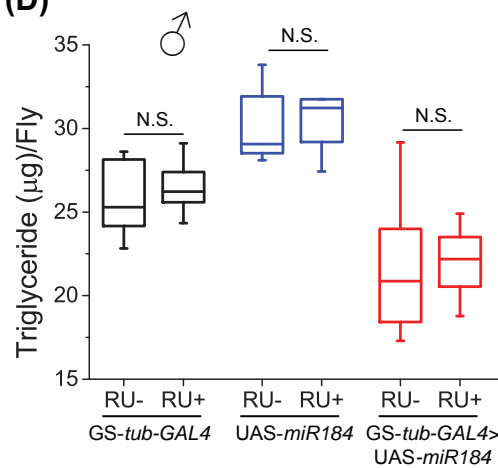

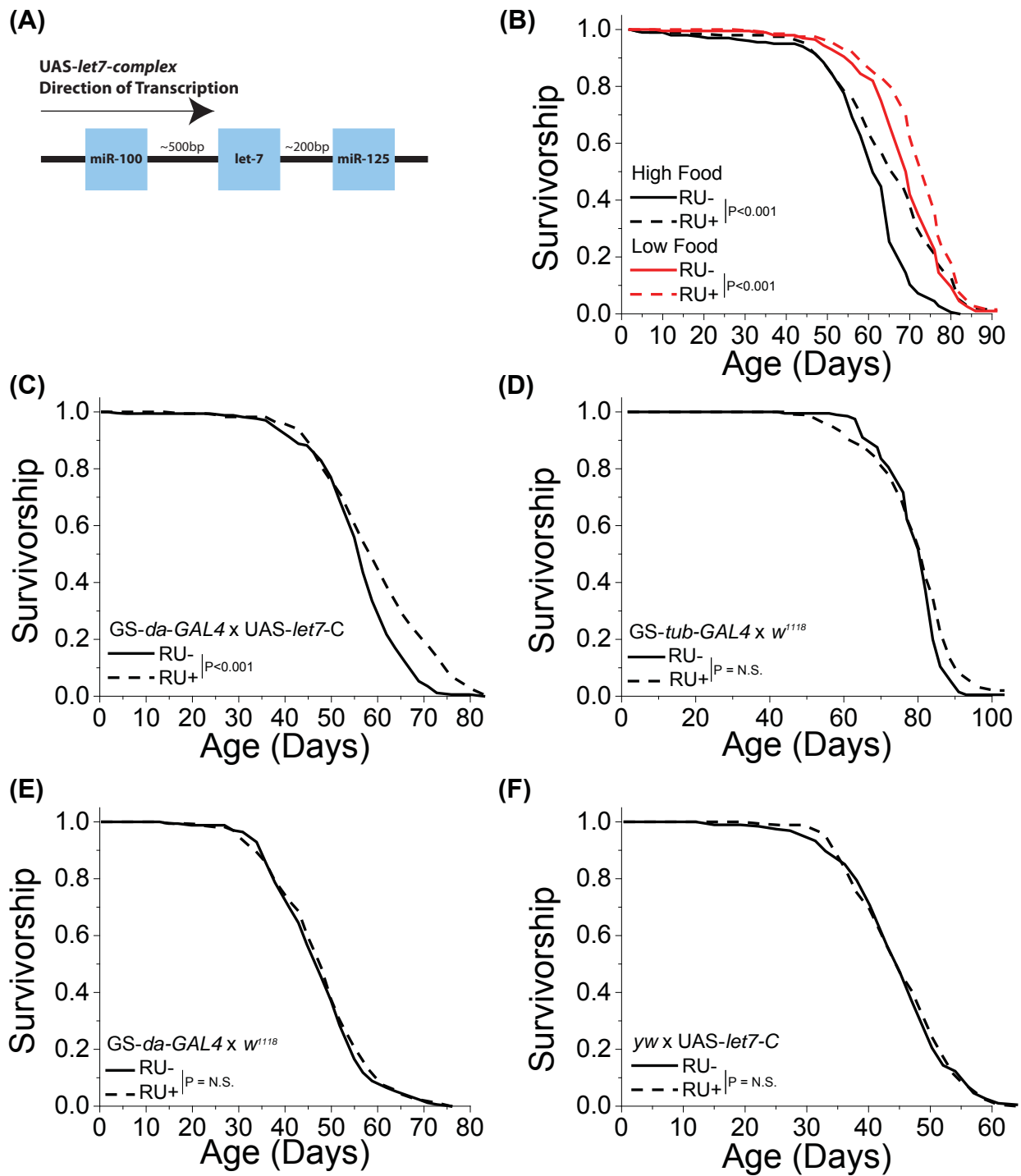

Supplemental Figure 2

(A)

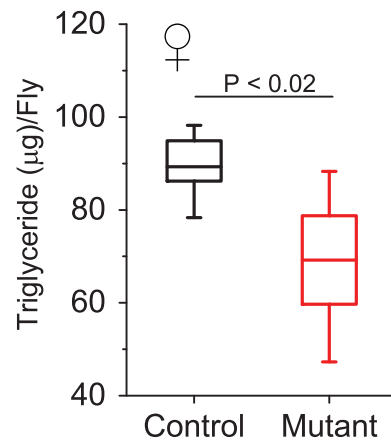

(B)

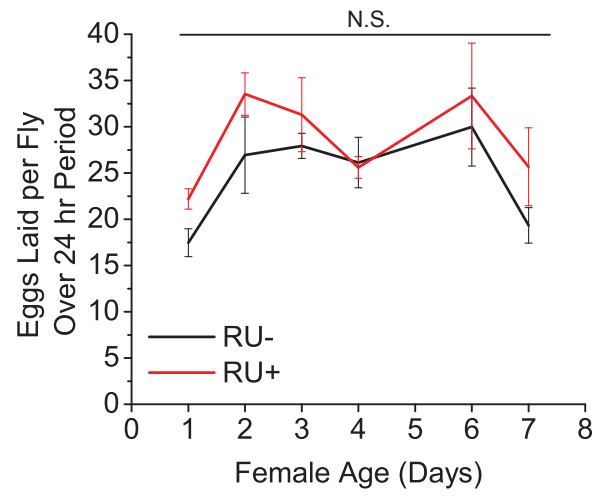

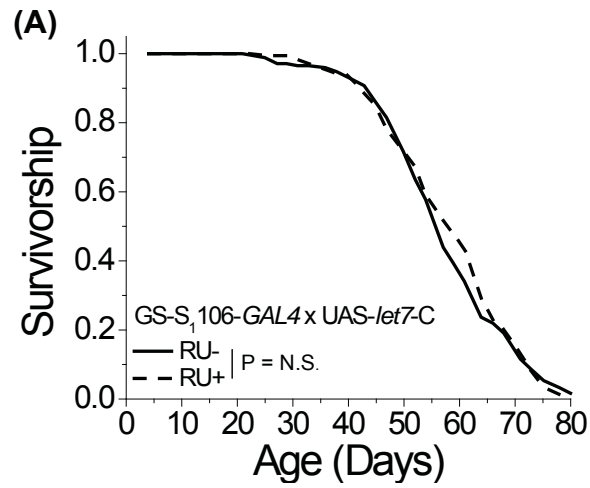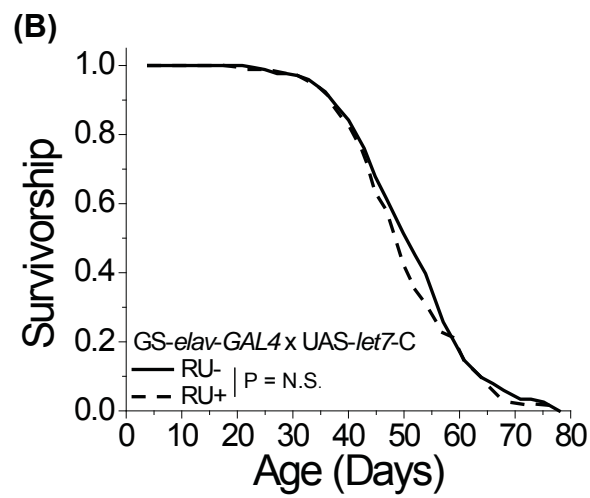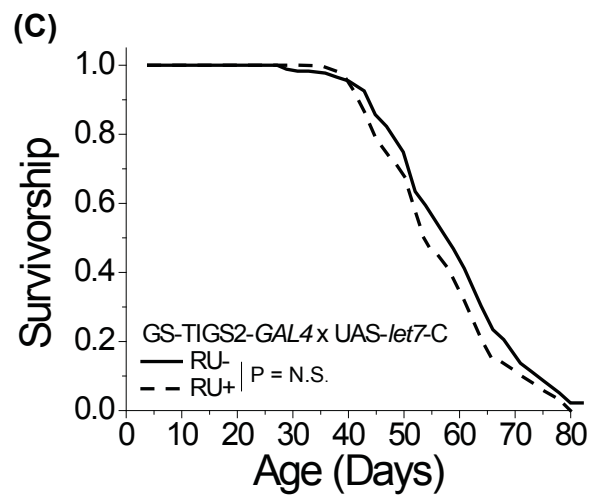

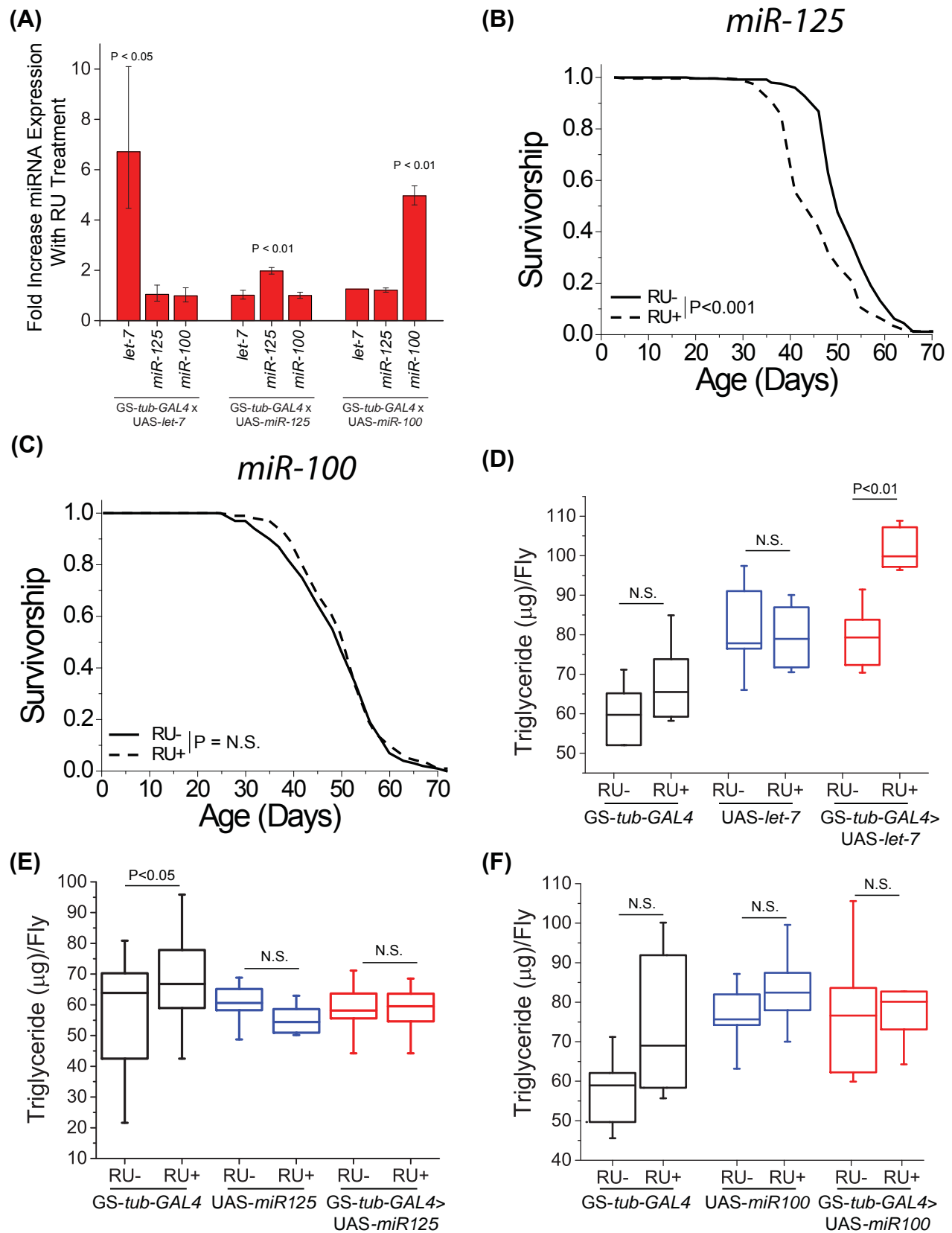

**Supplemental Figure 5**

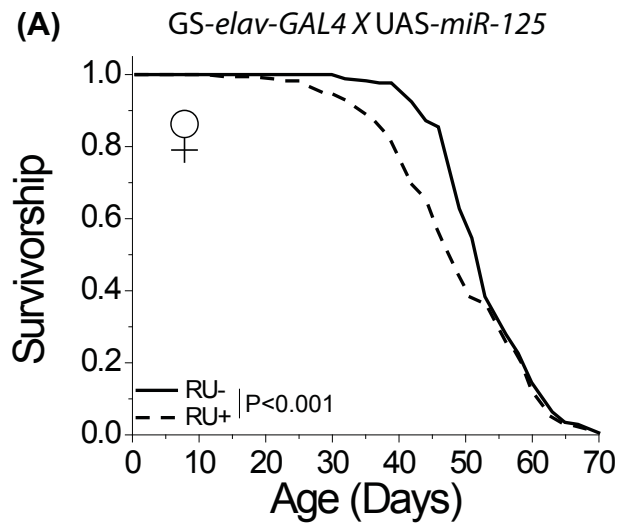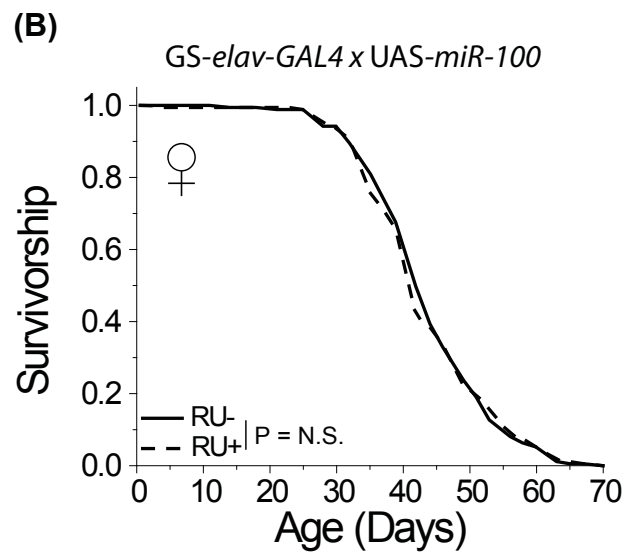

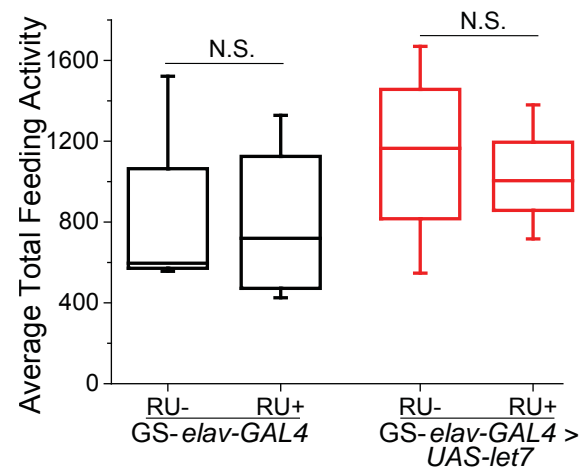

**Supplemental Figure 7**

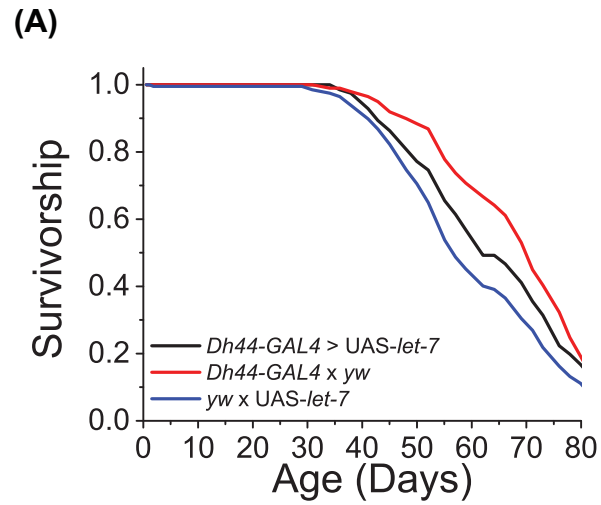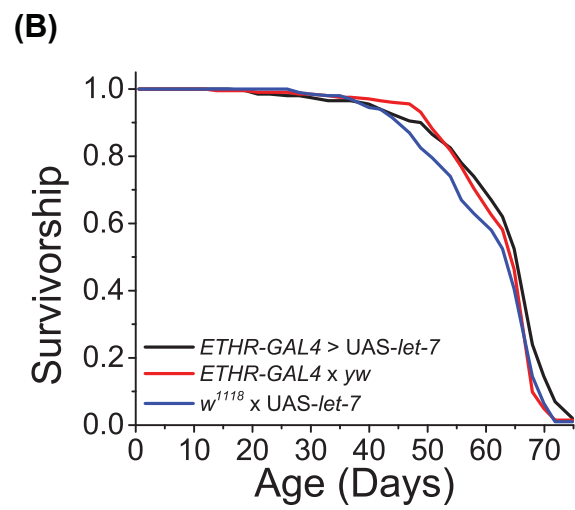

Supplement: Supplementary file 2 — Fig. S1 Identification of miRNA that are altered through diet, and analysis of miR‐184 overexpression flies. (A) Several miRNA are altered in flies when given either a high‐nutrient diet compared to those a low‐nutrient diet. Here, we highlight 4 miRNA that appeared to show some diet dependency: let‐7, miR‐8, miR‐34, and miR‐184. (B) qPCR of GS‐tubulin‐GAL4 > UAS‐miR‐184 flies show that feeding RU‐486 induces a 4‐fold increase in miR‐184 levels (N = 10 flies per food type). Ubiquitous overexpression of miR‐184 has no effect on TAG levels in females (C) or in males (D). In panel (C), N = 50 female flies for genotype/food treatment; in panel (D) N = 30 male flies for genotype/food treatment. Fig. S2 Adult‐specific let‐7‐C overexpression increases female lifespan, regardless of diet or type of ubiquitous driver. (A) Cartoon of the let‐7‐complex. All 3 miRNA of the let‐7‐C (miR‐100, let‐7, and miR‐125) are transcribed as a polycistronic mRNA molecule. (B) Adult‐specific let‐7‐C overexpression using the GS‐tubulin‐GAL4 driver significantly increases female fly lifespan, regardless of diet (N = 201 flies for high food RU‐, 197 flies for high food RU+, 198 flies for low food RU‐, and 199 flies for low food RU+). (C) Adult‐specific let‐7‐C overexpression using the GS‐daughterless‐GAL4 driver also significantly increases female fly lifespan (N = 170 flies for RU‐ food and 173 flies for RU+ food). Control crosses consisting of the GS‐tubulin‐GAL4 driver (D; N = 200 flies for RU‐ food and 196 flies for RU+ food), the GS‐daughterless‐GAL4 driver (E; N = 169 flies for both food types), or UAS‐let7‐C (F; N = 195 flies for RU‐ food and 185 flies for RU+ food), with or without RU‐486 feeding, has no significant effect on lifespan. Fig. S3 TAG is significantly decreased in let‐7‐C mutant female flies (A) and let‐7‐C overexpression has no effect on fecundity (B). In (A), N = 50 flies for both genotypes. (B) The number of eggs laid from female GS‐tubulin‐GAL4 > UAS‐let7‐C were counted [file ACEL-16-1434-s002.pdf]
